# Draft Genome Sequence of the Biocontrol and Plant Growth-Promoting Rhizobacterium ***Pseudomonas fluorescens*** strain UM270

**DOI:** 10.1186/s40793-015-0123-9

**Published:** 2016-01-13

**Authors:** Julie E. Hernández-Salmerón, Rocio Hernández-León, Ma. Del Carmen Orozco-Mosqueda, Eduardo Valencia-Cantero, Gabriel Moreno-Hagelsieb, Gustavo Santoyo

**Affiliations:** Instituto de Investigaciones Químico Biológicas, Universidad Michoacana de San Nicolás de Hidalgo, Morelia, Michoacán México; Department of Biology, Wilfrid Laurier University, Waterloo, Ontario Canada

**Keywords:** *Pseudomonas fluorescens*, Biocontrol, PGPR

## Abstract

The *Pseudomonas fluorescens* strain UM270 was isolated form the rhizosphere of wild *Medicago* spp. A previous work has shown that this pseudomonad isolate was able to produce diverse diffusible and volatile compounds involved in plant protection and growth promotion. Here, we present the draft genome sequence of the rhizobacterium *P. fluorescens* strain UM270. The sequence covers 6,047,974 bp of a single chromosome, with 62.66 % G + C content and no plasmids. Genome annotations predicted 5,509 genes, 5,396 coding genes, 59 RNA genes and 110 pseudogenes. Genome sequence analysis revealed the presence of genes involved in biological control and plant-growth promoting activities. We anticipate that the *P. fluorescens* strain UM270 genome will contribute insights about bacterial plant protection and beneficial properties through genomic comparisons among fluorescent pseudomonads.

## Introduction

Plant pathogens cause diverse crop plant diseases resulting in drastic economic losses around the world. An alternative to the use of chemicals to control plant pathogens is the employment of eco-friendly bacterial agents [1, 2]. An ideal bacterial biocontrol agent would be one with the additional capacity to directly stimulate plant growth [3]. Here, we report the draft genome sequence of the novel strain *Pseudomonas fluorescens* strain UM270. This strain was previously isolated and characterized for its excellent capacities for biocontrol of phytopathogens and plant growth promotion [4].

In a previous report, our group showed that the *P. fluorescens* strain UM270, among other three pseudomonad strains, was the best in promoting the growth of *Medicago truncatula* Gaertn. plants by significantly increasing biomass and chlorophyll content. During confrontation assays, strain UM270 inhibited the growth of agro-economically important fungal phytopathogens such as *Botrytis cinerea*, *Rhizoctonia solani**,**Diaporthe phaseolorum**,* and *Colletotrichum lindemuthianum* [4]. In biocontrol experiments, the strain UM270 protected *M. truncatula* plants from *B. cinerea* infection, reducing general stem disease symptoms, root browning and necrosis [4].

Importantly, the strain UM270 exerted these activities through the emission of either diffusible compounds (such as phenazines, cyanogens, 1-aminocyclopropane-1-carboxylate deaminase, siderophores, proteases and indole-3-acetic acid) or volatiles (like dimethyl disulfide and dimethylhexadecylamine) [4], revealing that the strain UM270 contains direct and indirect mechanisms to promote plant growth [5].

## Organism Information

### Classification and features

**Fig. 1:**
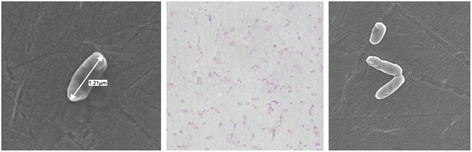
Images of *P. fluorescens* strain UM270 using scanning electron microscopy (left and right) and phase-contrast (center)

*P. fluorescens* strain UM270 is a Gram-negative, non-sporulating, motile, rod-shaped bacterium belonging to the Order *Pseudomonadales* and the Family *Pseudomonadaceae* (Fig. 1). The strain exhibits the general and common features of a *Pseudomonas* species phenotype (Table 1) [6].

**Table 1:** Classification and general features of *Pseudomonas fluorescens* strain UM270

MIGS ID	Property	Term	Evidence code^a^
	Current classification	Domain *Bacteria*	TAS [14]
		Phylum *Proteobacteria*	TAS [15]
		Class *Gammaproteobacteria*	TAS [16, 17]
		Order *Pseudomonadales*	TAS [18, 19]
		Family *Pseudomonadaceae*	TAS [18, 20]
		Genus *Pseudomonas*	TAS [18, 21]
		Species *Pseudomonas fluorescens*	TAS [18, 22]
		Strain UM270	TAS [4]
	Gram stain	Negative	TAS [6]
	Cell shape	Rod-shaped	TAS [6]
	Motility	Motile	NAS [6]
	Sporulation	None	NAS
	Temperature range	Mesophilic	IDA
	pH range; Optimum	6-8.5;7-8	IDA
	Optimum temperature	28 °C	IDA
	Carbon source	Heterotroph	IDA, [6]
	Energy source	Chemoorganotroph	NAS
MIGS-6	Habitat	Rhizospheric soil	TAS [4]
MIGS-6.3	Salinity	NaCl 1-4 %	IDA
MIGS-22	Oxygen Requirement	Aerobic	IDA
MIGS-15	Biotic relationship	*Medicago* spp. root associated	TAS [4]
MIGS-14	Pathogenicity	Non-pathogenic	TAS [4]
MIGS-4	Geographic location	Morelia, México	TAS [4]
MIGS-5	Sample collection	March, 2012	NAS
MIGS-4.1	Latitude	19° 46’ 6” N	TAS [4]
MIGS-4.2	Longitude	101° 11’ 22” W	TAS [4]
MIGS-4.3	Depth	10-20 cm	NAS
MIGS-4.4	Altitude	1800 M.A.S.L.	NAS

^a^Evidence codes - IDA: Inferred from Direct Assay; TAS: Traceable Author Statement (i.e., a direct report exists in the literature); NAS: Non-traceable Author Statement (i.e., not directly observed for the living, isolated sample, but based on a generally accepted property for the species, or anecdotal evidence). These evidence codes are from the Gene Ontology project

**Fig. 2:**
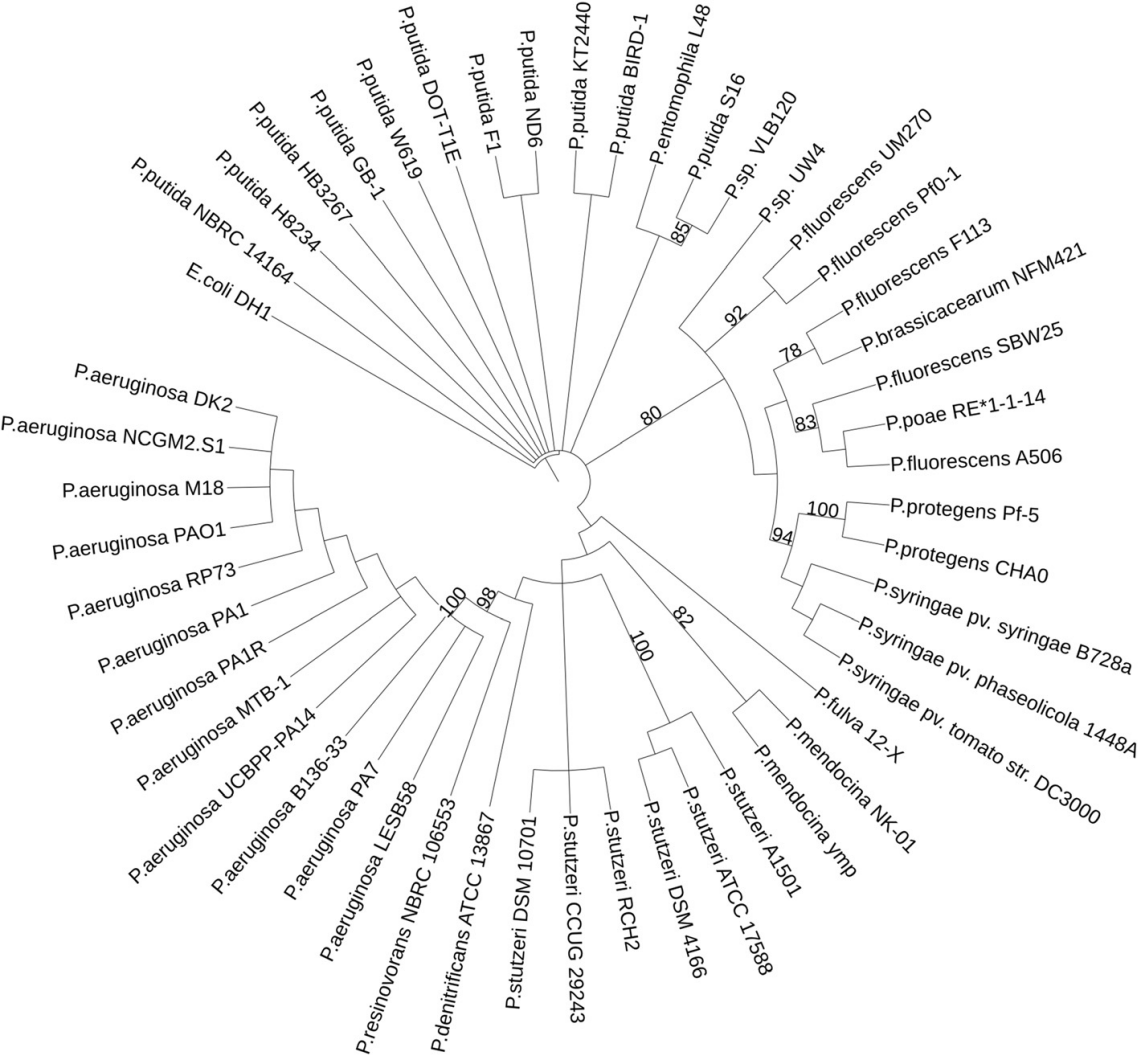
Phylogenetic tree showing the close relationship of *P. fluorescens* strain UM270 with *P. fluorescens* Pf0-1, as well as with other *Pseudomonas* species based on aligned sequences of the 16S rRNA gene. Phylogenetic analyses were performed using SeaView and edited in iTol. The tree was built using the maximum likelihood method. Bootstrap analysis (1000 replicates) was performed to assess the support of the clusters. *E. coli* was used as an outgroup

The UM270 strain was isolated from the rhizosphere of *Medicago* spp. located in an agricultural field in Morelia, Michoacán, México. As mentioned above, this bacterium was further characterized and found to produce several diffusible and volatile compounds involved in biocontrol against several fungal pathogens, particularly effective against the grey mold disease caused by *Botrytis cinerea* [4]. Recent work in our lab has demonstrated that this strain is highly competitive and an efficient root and rhizosphere colonizer, as well as an inducer of ISR (Induced systemic resistance) in plants [Rojas-Solis and Santoyo, Unpublished results]. The Minimum Information about the Genome Sequence of *P. fluorescens* strain UM270 is summarized in Table 1. Its phylogenetic position is shown in Fig. 2, where the 16S rRNA gene of *P. fluorescens* strain UM270 is 99 % similar to that of *P. fluorescens* strain Pf0-1 [7–9].

## Genome sequencing information

### Genome project history

The *P. fluorescens* strain UM270 was selected among other pseudomonads for its higher ability to control fungal pathogens and protect *Medicago truncatula* Gaertn. from *B. cinerea* infection [4], for being highly competitive, an excellent root and rhizosphere colonizer of maize plants and for inducing ISR in plants (Rojas-Solis and Santoyo, Unpublished results). A high-quality draft sequence of the genome has been deposited at DDBJ/EMBL/GenBank. A summary of the project information is shown in Table 2.

**Table 2:** Project information

MIGS ID	Property	Term
MIGS 31	Finishing quality	High-quality draft (Full genome representation)
MIGS-28	Libraries used	3 libraries of 400–450 bp, 600 bp and 1,000 bp.
MIGS 29	Sequencing platforms	Illumina MiSeq
MIGS 31.2	Fold coverage	45.0 ×
MIGS 30	Assemblers	Newbler v. 2.9
MIGS 32	Gene calling method	NCBI Prokaryotic Genome, Annotation Pipeline
	Locus Tag	RL74
	Genbank ID	JXNZ00000000
	GenBank Date of Release	2014-12-09
	GOLD ID	Gb0118948
	BIOPROJECT	PRJNA269735
MIGS 13	Source Material Identifier	UM270
	Project relevance	Agriculture, Plant-Bacteria Interaction, Biocontrol

### Growth conditions and genomic DNA preparation

From a single colony culture the *P. fluorescens* strain UM270 was inoculated on 50 ml of King’s B medium [10], grown overnight at 28 °C with in agitation (250 rpm). One milliliter of the culture was serially diluted to be analyzed further. We confirmed the morphology and antibiotic-resistance phenotype of the strain. From the culture, 20 ml were taken to isolate the genomic DNA by using the Wizard® Genomic DNA Purification Kit following manufacture’s instructions (Promega). DNA samples were subjected to an additional purification step with the same Wizard® Genomic DNA Purification Kit (Promega). The quality and quantity of the final DNA sample were evaluated by agarose gel electrophoresis and by using a NanoDrop 1000 Spectrophotometer (Thermo Scientific).

### Genome sequencing and assembly

Genomic DNA samples of *P. fluorescens* strain UM270 were sent to a sequencing service at the LANGEBIO-Irapuato, México. Genome sequencing was performed using a MiSeq Sequencer (Illumina, Inc.) generating three paired-end libraries (400–450 bp, 600 bp and 1,000 bp, respectively) with a coverage of approximately 45×. The *P. fluorescens* strain UM270 draft genome we ran a blastn comparison using the contigs as query, against the genome sequence of *P. fluorescens* Pf0-1 as target reference. To order the contigs we followed the matching coordinates of the reference genome. Project information is shown in Table 2.

### Genome annotation

Genome annotation was carried out with RAST [11] and the Prokaryotic Genome Annotation Pipeline tools [12]. Statistics for the genome assembly were calculated using software Newbler v2.9 (Roche) and are shown in Table 2. This Whole Genome Shotgun sequence project has been deposited at DDBJ/EMBL/GenBank under accession JXNZ00000000. The version described in this paper is version JXNZ00000000.

## Genome Properties

**Fig. 3:**
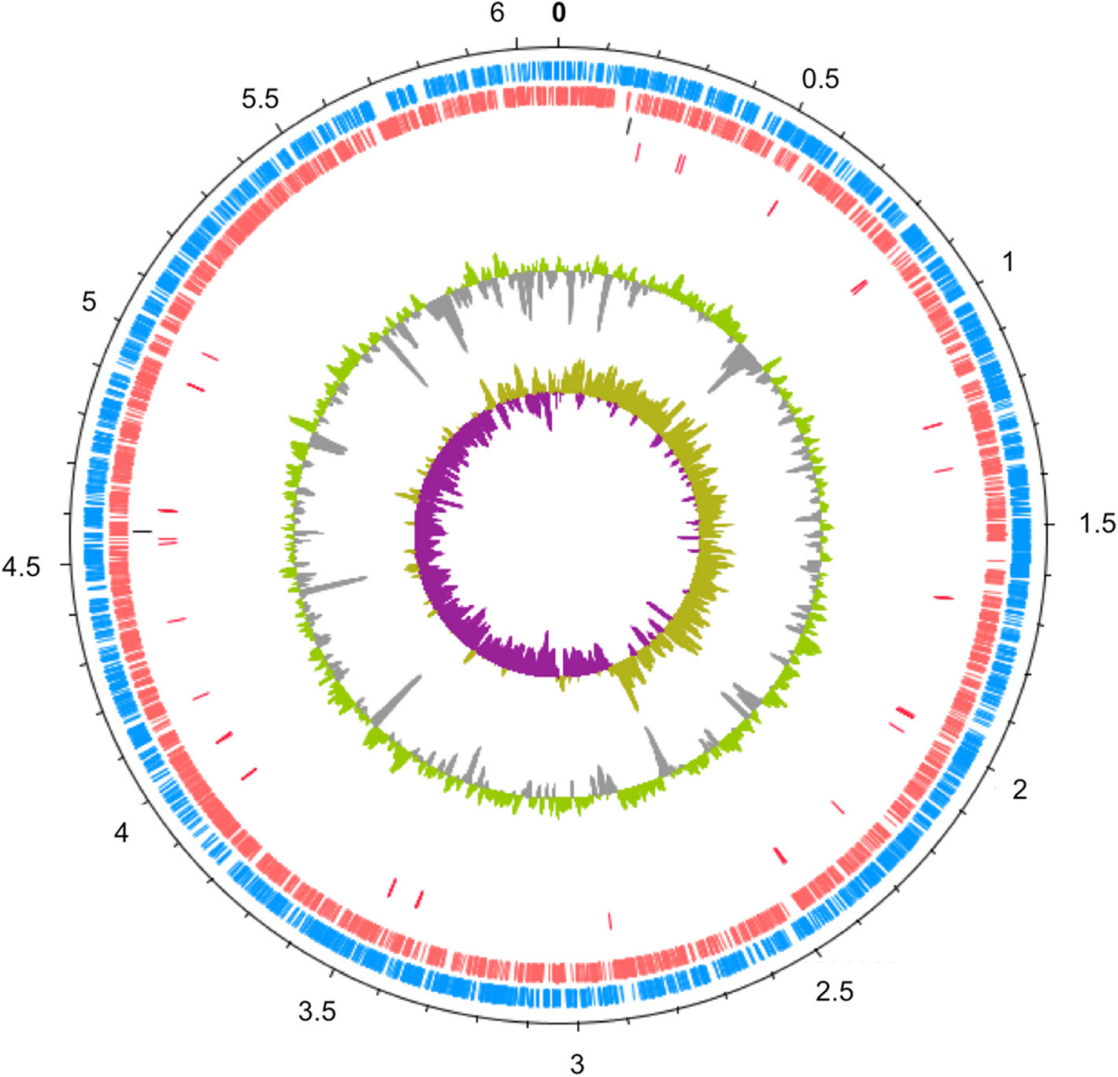
Graphical map of the *P. fluorescens* strain UM270. Numbers represent Megabases (Mb). From outside to the center: Genes on forward strand (blue), Genes on reverse strand (red), RNA genes (rRNAs black color, tRNAs red color) G + C% (green and gray), G + C skew (purple and yellow). To display the *P. fluorescens* strain UM270 draft genome we ran a blastn comparison using the contigs as query, against the genome sequence of *P. fluorescens* strain Pf0-1 as target reference. We then used these results to order the contigs following the matching coordinates of the reference genome. Contigs not matching the reference genome were ordered from largest to smallest and appended to the contigs matching the genome of reference. The ordered contigs were joined with 50 bp of “N” to draw this figure using the DNA plotter software

The total length of the assembled sequences obtained was 6,047,974 bp belonging to one chromosome, with a G + C content of 62.66 %. The sequenced fragments of the genome are predicted to contain 5,509 genes, consisting of 5,396 coding sequences, 59 RNA genes, 110 pseudogenes and 14 frameshifted genes. Genome statistics are in Table 3 and a graphical map is represented in Fig. 3. The Table 4 presents the number of genes associated with the COG functional categories.

**Table 3:** Genome statistics

Attribute	Value	% of total
Genome size (bp)	6,047,974	100.00
DNA coding (bp)	5,284,158	87.00
DNA G + C (bp)	3,772,331	62.00
DNA scaffolds	524	100.00
Total genes	5,509	100.00
Protein coding genes	5,396	98.00
RNA genes	59	-
Pseudo genes	110	1.90
Genes in internal clusters	NA	-
Genes with function prediction	4,490	82.00
Genes assigned to COGs	3,821	68.00
Genes with Pfam domains	4,297	78.00
Genes with signal peptides	5	0.09
Genes with transmembrane helices	30	0.50
CRISPR repeats	0	-

**Table 4:** Number of genes associated with the 25 general COG functional categories

Code	Value	% of total^a^	Description
J	159	2.94	Translation, ribosomal structure and biogenesis
A	0	0.00	RNA processing and modification
K	342	6.33	Transcription
L	117	2.16	Replication, recombination and repair
B	3	0.00	Chromatin structure and dynamics
D	32	0.59	Cell cycle control, cell division, chromosome partitioning
Y	0	0.00	Nuclear structure
V	55	1.01	Defense mechanisms
T	216	4.00	Signal transduction mechanisms
M	212	3.92	Cell wall/membrane biogenesis
N	142	2.63	Cell motility
Z	0	0.00	Cytoskeleton
W	0	0.00	Extracellular structures
U	55	1.01	Intracellular trafficking and secretion
O	150	2.77	Posttranslational modification, protein turnover, chaperones
C	244	4.52	Energy production and conversion
G	190	3.52	Carbohydrate transport and metabolism
E	434	8.04	Amino acid transport and metabolism
F	78	1.44	Nucleotide transport and metabolism
H	143	2.65	Coenzyme transport and metabolism
I	185	3.42	Lipid transport and metabolism
P	226	4.18	Inorganic ion transport and metabolism
Q	67	1.24	Secondary metabolites biosynthesis, transport and catabolism
R	364	6.74	General function prediction only
S	372	6.89	Function unknown
-	1,610	29.83	Not in COGs

^a^The total is based on the total number of protein coding genes in the annotated genome

## Insights from the genome sequence

The draft genome sequence reported here covers its full genome and at first analysis reveals the presence of multiple genes participating in the synthesis of diffusible metabolites and volatile organic compounds produced by *P. fluorescens* strain UM270. Some of this antimicrobial arsenal includes compounds like phenazine (*phzFABCD*), pyocyanin (*pcnCDE*), pyoverdine (*pvdPD*), 2,4-diacetylphloroglucinol (*phlACBD*) and the volatile hydrogen cyanide (*hcnCB*), important for the biological control of several plant diseases caused by phytopathogenic fungi, oomycetes, and bacteria [2]. Other plant-bacteria communication genes detected in the strain UM270 genome are *acdS* and *iaaMH*, encoding for an ACC deaminase (1-aminocyclopropane-1-carboxylate) protein and IAA (indole-3-acetic acid) biosynthesis. The synergistic interaction of ACC deaminase and both plant and bacterial auxin, IAA, is relevant for the optimal functioning of PGPR to directly promote plant growth and also protect plants against environmental stresses, and bacterial and fungal pathogens [5]. Other genes such as *pcdQ*, which codes for an Acyl-homoserine lactone acylase, important for bacterial communication and biofilm formation, were detected, as well as Secretion Systems Type II to VI and orthologs of the toxin-antitoxin loci *vapBC-1* and *vapXD*. These last determinants are important for survival, competence and colonization of the rhizosphere and root systems [13].

## Conclusions

The strain UM270 was selected for genome sequencing due to its biocontrol and plant growth promoting properties [4]. The plant beneficial mechanisms exerted by this rhizobacterium involved direct and indirect mechanisms. Here, the draft genome sequence of the *P. fluorescens* strain UM270 revealed further genetic elements involved in plant-bacterial communication, as well as in rhizosphere competence and colonization. We anticipate that the genome of *P. fluorescens* strain UM270 will contribute to new insights about biocontrol and plant beneficial activities through genomic comparisons among available complete genomes of pseudomonad strains.
